# Prevalence of intestinal parasites and associated risk factors among inmates of Mekelle prison, Tigrai Region, Northern Ethiopia, 2017

**DOI:** 10.1186/s12879-019-4053-9

**Published:** 2019-05-10

**Authors:** Fitsum Mardu, Mekonnen Yohannes, Desalegn Tadesse

**Affiliations:** 1 Department of Medical Laboratory Sciences, College of Medicine and Health Sciences, Adigrat University, Adigrat, Ethiopia; 2 Unit of Medical parasitology and entomology, Institute of Biomedical Sciences, Mekelle University, Mekelle, Ethiopia

**Keywords:** Intestinal parasites, Prison inmates, Mekelle prison, Prevalence, Tigrai, Ethiopia

## Abstract

**Background:**

In Ethiopia, like other developing countries, intestinal parasitic infections are the major public health problems affecting millions annually. Overcrowding and poor living conditions are the major risk factors. Prison inmates are among the most vulnerable groups to intestinal parasitic infections. However, there is scarcity of epidemiological data regarding intestinal parasites among prison inmates in Ethiopia, notably in Tigrai. Thus, we aimed to determine the prevalence of intestinal parasites and identify the associated factors among inmates of Mekelle prison, Tigrai, Northern Ethiopia.

**Methods:**

A cross sectional study involving 291 inmates was conducted from February to June 2017 among inmates of Mekelle prison. After systematically selecting subjects, stool specimens were examined using direct wet mount and formol-ether concentration techniques. We used SPSS version 21 for data analysis. We considered *p*-value less than 0.05 significant at 95% confidence level.

**Results:**

Of the 291 inmates enrolled in the study, 124 (42.6%) harbored one or more intestinal parasites. The protozoan *Entamoeba histolytica/dispar/moshkovskii* was the predominant parasite accounted for 68 (23.3%) of the infections followed by *Giardia lamblia* (10.3%) and *Entamoeba coli* (8.2%). Fourteen (4.8%) participants were co-infected with different parasite species. The co-infections of *Entamoeba histolytica/dispar/moshkovskii* and *Giardia lamblia* were detected among 3.1% of the participants. In bivariate analysis, hand fingernail status (COR 1.86, 95% CI, 1.08–3.20) and duration of stay in prison (COR 2.23, 95% CI 1.31–3.79) were statistically associated with intestinal parasite infections. In multivariable regression, inmates who stayed in the prison for one year or less were more likely to harbor intestinal parasitic infections (*p* = 0.013) than those who stayed longer. No other single predictor variable was found to be significantly associated with intestinal parasitic infections.

**Conclusions:**

The result of this study showed that intestinal parasites are significant health problems among inmates of Mekelle prison.

## Background

Intestinal parasitic infections contribute to high global health burden causing over 3.5 billion infections as well as clinical morbidity of 450 million, the majority being vulnerable groups in developing countries [1]. It is estimated that over 2 billion people worldwide are infected with soil-transmitted helminthes (STHs). STHs are also responsible for the rate of Disability Adjusted Life Years (DALYs) of approximately 39 million, indicating a substantial economic burden of these infections. Among these parasites, *Ascaris lumbricoides* infects over a billion people while *Trichuris trichiura* and hookworms infect over 795 million and 740 million people worldwide, respectively [2].

Schistosomiasis affects over 240 million people, and more than 700 million are at risk of infection globally. It is prevalent in tropical and sub-tropical areas where poverty, lack of potable water, and inadequate sanitation prevail. Besides, more than 200,000 annual deaths in Sub-Saharan Africa (SSA) are attributable to schistosomiasis. In 2015, more than 218 million people required treatment against schistosomiasis: of which 90% live in Africa [3]. Among the intestinal protozoa, *Giardia lamblia* infects over 200 million people worldwide with 50,000 annual incidences. *Entamoeba histolytica* causes hundreds of millions of infections each year; approximately 50 million of these suffer from severe morbidity resulting in an estimated 100,000 deaths each year [4].

Intestinal parasitic infections (IPIs) are considered as infectious diseases of poverty. They are closely related with low household income, poor sanitation, limited access to clean water, tropical climate, low altitude, and overcrowding [5]. They cause significant morbidity and mortality in low and middle-income countries [6]. Some of the IPIs have also been associated with complications of other severe diseases such as HIV, tuberculosis, and malaria [7, 8]. Malnutrition, protein deficiency, increment in health costs, cognitive impairment, absence from work, diagnostic and treatment expenses have also been attributable to these infections [9].

In Ethiopia, like other developing countries, intestinal parasites are major public health problems being the first or second most predominant causes of outpatient morbidity [10]. STHs for example are widely distributed in the country with 81 million people living in endemic areas: 9.1 million preschool children, 25.3 million schoolchildren, and 44.6 million adults. The number of individuals living in areas targeted for treatment of STHs is 56.7 million: among which 31.32 million are adults. Currently, 475 woredas (districts) in Ethiopia require treatment against these infections [11].

Prison inmates are among the vulnerable groups to intestinal parasitic infections. In developing countries, prison inmates live in deprived situations characterized by inadequate facilities, malnutrition, scarce potable water, over-crowdedness, and poor hygiene [12]. Besides this, prisoners have no control of their environment in which they live, which pose them at risk of infection with intestinal parasites [13]. An estimated more than10 million people are in prisons worldwide; the majority being in developing countries particularly in SSA where health systems are inadequate [14, 15]. Prisoners harbor diseases that are determined both by the environment from which they come and by the prison in which they live. Because of this and other reasons, prison inmates carry a much burden of illness than other members of the community [16].

Thus, there is a dear need to continuous screening of endemic communities to reduce the burden of IPIs. WHO recommends periodic de-worming of intestinal parasites twice a year if prevalence is over 50% and once if prevalence exceeds 20% [17]. Renewed and up-to-date information on the epidemiology of IPIs in more vulnerable groups such as prisoners may significantly contribute towards improving the health condition of such at-risk groups. Thus, we aimed to provide epidemiological data on the prevalence of intestinal parasites and associated factors among inmates of Mekelle prison, Northern Ethiopia.

## Methods

### Study setting

We conducted a cross sectional study to determine the prevalence of intestinal parasites and associated risk factors among inmates of Mekelle prison from February to June 2017. Mekelle, the capital city of Tigrai regional state, is located 780 kms North of Addis Ababa (the capital of Ethiopia) at a latitude and longitude of 13°29°N 39°28°E. The city is characterized by relatively high temperature (above 18^o^c) and evenly distributed precipitation throughout the year. It is elevated 2254 m above sea level, close to the edge of the northern portion of the Ethiopian Rift Valley in a semi-arid area with a mean annual rainfall of 714 mm [18]. According to Central Statistics Agency of Ethiopia (2016), Mekelle has a total population of 310,436. The largest proportion of the population in the city depends on government employment, commerce, and small-scale enterprises. Administratively, Mekelle is considered a special zone, which is divided into seven sub-cities. It is the economic, cultural, and political hub of northern Ethiopia [19]. With regard to health facilities, there is one Comprehensive Specialized Referral Hospital (Ayder), Three General Hospitals (Mekelle, Semen Ez, and Quiha General Hospitals), and Seven Health Centers (Quiha, Aynalem, Kassech, Serawat, Semen, Mekelle, and Adishumdihun). Mekelle prison, found within Mekelle city, houses 2541 inmates during the study period: of these 97.5% (2478) were males while the rest 2.5% (63) were females. The prisoners represent different age groups, ethnicity, educational attainment, as well as occupational backgrounds. They were provided with education, skill trainings, and opportunities to access finance and engage in economically useful activities.

### Study population and eligibility criteria

Our source population was all prisoners in Mekelle prison available during the study period. Those inmates with or without gastro-intestinal symptoms were enrolled in the study. We excluded inmates who took treatment against intestinal parasites within 1 month of study time and those who were critically ill or mentally disabled (unable to bring sample or respond to questions).

### Sample size and sampling technique

We used single population proportion formula (*n* = Z^2^_α/2*_p (1-p) / d^2^) to calculate the sample size. At 95% confidence level, expected population proportion (p), margin of error (d), and critical value for confidence interval (Z); the sample size was calculated to be 301 after adding 10% non-response rate. The proportion ‘p’ is 0.73, taken from previous prevalence (72.7%) of intestinal parasites among inmates of Shewa-Robit prison, north central Ethiopia [17]. We finally recruited participants using systematic random sampling technique.

### Data collection and quality control

After explaining the purpose and relevance of the study, participants were asked for their consent. Socio-demographic data were collected by interviewing study participants using structured questionnaire. Those participants who were able to read and write filled the questionnaire in their own and had an opportunity to ask any questions. To ensure quality of data, the data collectors had been trained before data collection started. We also oriented the participants on proper sample collection. The quality of laboratory analysis was maintained by following standard operating procedures during pre-analytical, analytical, and post-analytical stages.

### Sample collection and examination

Study participants were instructed to bring about 2–3 g of stool specimens (using roll of cotton as reference) with pre-labeled, leak-proof, plastic containers. After checking the quality and quantity of each sample, direct wet mount was performed at the laboratory section in the prison. Then, all specimens were preserved in 10% formalin and transported to Medical University Parasitology Laboratory; where we performed formol-ether concentration technique. Lugol’s iodine solution was used for the identification of cysts of intestinal protozoan parasites.

### Data analysis

We analyzed our data using SPSS version 21 software. Frequency distributions and percentages of variables were calculated. Furthermore, bivariate and multivariate logistic regressions were performed to determine the association between the independent and outcome variables. We declared statistical significance when *p*-value is less than 0.05 at 95% confidence interval.

## Results

### Socio-demographics of study participants

From the total sample size (301) proposed, 291 (96.7%) inmates volunteered to participate in the study. Majority, 285(97.9%) of the study participants were males. Age of the study subjects ranged from 15 to 78 years with a mean age of 30.38 years. About 39% of the study participants were found within the age group of 25–34 years. Moreover, 54.6% of the subjects responded that they were urban residents before imprisonment (Table 1).

**Table 1:** Socio-demographic profile and prevalence of intestinal parasites among inmates of Mekelle prison, Northern Ethiopia, 2017

Variables	Frequency (%)	Positive for IPIs	
		Number	Percent
Age groups (years)			
15-24	101 (34.7)	44	15.1
25-34	113 (38.8)	44	15.1
35-44	44 (15.1)	22	7.5
≥45	33 (11.3)	14	4.8
Sex			
Male	285 (97.9)	120	41.2
Female	6 (2.1)	4	1.4
Education level			
No formal education	36 (12.4)	15	5.1
Primary school	93 (32)	46	15.8
Secondary school	132 (45.4)	52	17.9
College/university	30 (10.3)	11	3.8
Residence before prison			
rural	132 (45.4)	58	20
urban	159 (54.6)	66	22.6
Occupation before imprisonment			
farmer	90 (30.9)	42	14.4
merchant	29 (10)	16	5.5
student	46 (15.8)	11	3.8
employee	41 (14.1)	14	4.8
others	85 (29.2)	41	14
Duration in prison			
≤ one year	77 (26.4)	44	15.1
> one year	214 (73.6)	80	27.5
Total	291	124	42.6

### Prevalence of intestinal parasites with respect to socio-demographics

The overall prevalence of intestinal parasites among the participants was 42.6%. The most affected age groups were 15–24 and 25–34 years with the same prevalence of 15.1%. Similarly, male participants were more affected (41.2%) than females (1.4%). In addition, those participants who stayed in the prison for more than 1 year had high proportion of intestinal parasitic infections (27.5%) compared to those who stayed for 1 year or less (15.1%). Moreover, the prevalence of intestinal parasites was high (14.4%) among inmates who were farmers before imprisonment. Socio-demographic background and prevalence of intestinal parasites among the participants are summarized in Table 1.

### Species of intestinal parasites identified

One hundred and 24 of the participants (42.6%) were infected with at least one parasite species. The protozoan *E. histolytica/dispar/moshkovskii* was the predominant parasite: accounted for 68 (23.3%) of the infections followed by *G. lamblia* 30 (10.3%), and *E. coli* (8.2%). Among the common intestinal helminthes were Taenia species and hookworm species detected among Eight (2.7%) and Six (2%) participants, respectively. Table 2 shows single and co-infections of intestinal parasites identified among the study participants.

**Table 2:** single and co-infections of intestinal parasites detected among study participants of Mekelle prison, Northern Ethiopia, 2017

Species of parasites	Number (*n* = 291)	Percent
Single infections		
*E. histolytica/dispar/moshkovskii*	55	18.9
*E. coli*	23	7.9
*G. lamblia*	20	6.8
Taenia species	7	2.4
Hookworm	5	1.7
Total	110	37.8
Co-infections		
*E. histolytica/E. dispar/E. moshkovskii* + *G. lamblia*	9	3.1
*E. histolytica/ E. dispar/E. moshkovskii* + *E. vermicularis*	1	0.34
*E. histolytica/E. dispar/ E. moshkovskii* + *S. mansoni*	1	0.34
*E. histolytica/E. dispar/ E. moshkovskii* + Taenia species	1	0.34
*G. lamblia* + *E. coli*	1	0.34
*E. histolytica/E. dispar/ E. moshkovskii + T. trichiura +* Hookworm	1	0.34
Total	14	4.8

Fourteen (4.8%) of the infected participants were co-infected with at least two intestinal parasites. The co-infection of *E. histolytica/dispar/moshkovskii* and *G. lamblia* were identified among Nine (3.1%) of the participants. Moreover, one inmate harbored the triple infection of *E. histolytica/dispar/moshkovskii*, Hookworm, and *T. trichiura* (Table 2). Prevalence of intestinal protozoa was 38.8%, and that of intestinal helminthes was 5%. The co-infection of intestinal protozoa and intestinal helminthes was reported in 1.4% of the study participants.

### Factors associated with intestinal parasitic infections

Majority of the participants (113, 38.8%) were found within the age group of 25–34 years; of which 38.9% (44/113) were infected with intestinal parasites. The differences in intestinal parasite distributions among age groups was not statistically significant (*p* > 0.05). In addition, 41.2% (120/291) of the male and 1.37% (4/291) of the female participants harbored intestinal parasites. Moreover, 54.6% of the study participants reported that they were urban residents before imprisonment; of these, 41.5% harbored one or more intestinal parasites. This difference in intestinal parasite prevalence among inmates from rural and urban areas was not significant (COR0.9, 95% CI 0.56–1.44). The bivariate and multivariable analysis of factors associated with intestinal parasites is presented in Table 3.

**Table 3:** Bivariate and multivariable analysis of factors associated with intestinal parasites among inmates of Mekelle prison, Northern Ethiopia, 2017

Variables	Intestinal parasites		COR (95% CI)	AOR (95% CI)	*p*-value
	Positive (%)	Negative (%)			
Gender^b^					
Male	120 (42.1)	165 (57.9)	-		-
Female	4 (66.7)	2 (33.3)			
Age groups					
15-24	44 (43.6)	57 (56.4)	1.03 (0.46-2.29)	-	-
25-34	44 (38.9)	69 (61.1)	1.03 (0.47-2.26)		
35-44	22 (50)	22 (50)	0.80 (0.32-2.00)		
≥ 45	14 (42.4)	19 (57.6)	1		
Education level					
Illiterate	15 (41.7)	21 (58.3)	0.81 (0.31-2.1)	-	-
Primary school	46 (49.5)	47 (50.5)	0.59 (0.25-1.3)		
Secondary school	52 (39.4)	80 (60.6)	0.89 (0.39-2.0)		
College/university	11 (36.7)	19 (63.3)	1		
Residence before imprisonment					
Rural	58 (43.9)	74 (56.1)	0.90 (0.56-1.44)	-	-
Urban	66 (41.5)	93 (58.5)	1		
Occupation before imprisonment					
Employed	14 (34.1)	27 (65.9)	1		
Merchant	16 (55.2)	13 (44.8)	0.42 (0.16-1.12)		
Student	11 (23.9)	35 (76.1)	1.65 (0.65-4.20)		
Farmer	42 (46.7)	48 (53.3)	0.59 (0.27-1.27)		
Others	41 (48.2)	44 (51.8)	0.55 (0.25-1.20)	-	-
Duration in prison					
≤ 1 year	44 (57.1)	33 (42.9)	2.23 (1.31- 3.79)^a^	2.0 (1.16-3.47)^a^	0.013
> 1 year	80 (37.4)	134 (62.6)	1	1	
Finger nail status					
Trimmed	86 (38.9)	135 (61.1)	1		
Untrimmed	38(54.3)	32 (45.7)	1.864 (1.08-3.20)^a^	1.56 (0.88-2.75)	0.123
Hand washing after toilet					
always	118(42.6)	159(57.4)	1	-	-
sometimes	6(42.9)	8(57.1)	0.99 (0.33-2.92)		
Wash hands with					
Water & soap	54(43.2)	71(56.8)	1	-	-
Water only	64(42.1)	88(57.9)	1.04 (0.64-1.68)		
Hand washing before meal^b^					
Always	121(42.6)	163(57.4)	-	-	-
Some times	3(42.9)	4(57.1)			

*COR* Crude odds ratio

*AOR* Adjusted odds ratio

*CI* Confidence interval

*1* referent

^a^significant association

^b^sample size for females and participants who sometimes wash their hands before meal were too small to perform any statistical test

Ninety (31%) of the study participants responded that they were farmers before imprisonment: of which, 46.7% (42/90) were infected with intestinal parasites. The distribution of intestinal parasites was not significantly associated (*p* > 0.05) with occupation before imprisonment in bivariate analysis. On the other hand, 54.3% (38/70) of the inmates with untrimmed hand fingernails were positive for intestinal parasites which is higher relative to those with trimmed hand finger nails (38.9%, 86/221). This difference was statistically significant (COR 1.864, 95% CI 1.08–3.20). Likewise, majority (51.9%) of the participants who stayed in the prison for 1 year or less had higher proportion of intestinal parasite compared to those who stayed longer. This difference in intestinal parasite with duration of stay in prison was statistically significant (COR = 2.23, 95% CI = 1.31–3.79) in bivariate analysis.

Factors with *p*-value less than 0.05 in bivariate analysis were further analyzed by multivariate regression analysis model. Therefore, after adjustments for other variables, inmates who stayed in the prison for 1 year or less were more likely to harbor intestinal parasites than those who stayed longer (*p* = 0.013). No other single predictor variable was found to be significantly associated with intestinal parasitic infections among the participants (Table 3).

## Discussions

The study aimed to determine the prevalence of intestinal parasites among inmates of Mekelle prison, northern Ethiopia. Accordingly, 42.6% of the participants were infected with at least one intestinal parasite species. The prevalence in the present study is lower than other studies conducted in Ethiopia and other prisons in Africa [17, 20, 21], but higher compared to a study conducted among inmates of Kisii prison, Kenya with prevalence of 24.7% [22]. On the other hand, the prevalence in this study is comparable with the report from Omdurman prison, Sudan, which revealed an overall prevalence of 49% [23]. The variations in the prevalence of intestinal parasites between the different prisons may be attributable to the difference in the number of participants, sanitary condition of prison services, laboratory techniques applied, climatic as well as environmental conditions, and study designs employed to carry out the studies.

The burden of intestinal protozoa in this study (38.8%) is higher than that of intestinal helminthes (5%). This supports a report from inmates of Ouagadougou prison, Burkina Faso, [21] and Jos central prison, Nigeria, [13]. The observed shortage of drinking water supply, poor sanitary condition of the toilets and cells, unavailability of soaps for hand washing might have contributed for the high prevalence of intestinal protozoa in our study. The difference in mode of transmission of intestinal protozoa and intestinal helminthes might also attribute to the differences. For example, infection of hookworm and *S. stercoralis* need contact of exposed skin with contaminated soil while *S. mansoni* is acquired by cercarial skin penetration during contact with infested water. Therefore, as prisoners are confined in the prison having limited activities, they have low chance of acquiring these intestinal helminthic infections.

On the other hand, intestinal protozoa are directly transmitted through feco-oral route while some intestinal helminthes (such as *T. trichiura* and *A. lumbricoides*) require a period of maturation in the environment. In this regard, auto-infections of intestinal protozoa among the inmates might not be uncommon. Besides, intestinal protozoa multiply within human hosts as opposed to helminthes. This may caused intestinal helminthes undetectable when infecting in very few numbers. However, the reasons for the higher prevalence of intestinal helminthes compared to protozoan parasites reported from studies such as Maiduguri prison, North Eastern Nigeria [24] remain unclear.

In Ethiopia, like in other developing countries, it is generally unknown whether the amoebic infections reported are due to the non-invasive *E. dispar/E. moshkovskii* or the invasive *E. histolytica* [25]. In the present study, the protozoan *E. histolytica/dispar/moshkovskii* was the commonly encountered intestinal parasite (23.3%) followed by *G. lamblia* (10.3%) and *E. coli* (8.2%). The dominance of *E. histolytica/dispar/moshkovskii* in this study agreed with other studies conducted in prisons [23] and in the general population [26]. Whether the reported *Entamoeba* species is the invasive or the non-invasive type, the mere presence of these parasites along with *G. lamblia* and *E. coli* indicates unhygienic situations in the prison. Although the analysis of the present study does not imply any prison situation as a risk factor, the observed poor sanitary conditions of the toilets and cells, accompanied by shortage of water in the prison may explain the common occurrence of intestinal protozoa in the study area. Taenia species and the hookworms are amongst the common intestinal helminthes detected among the study participants. Taenia species, which is common in Ethiopia, is mainly transmitted to humans via ingestion of raw/undercooked meat containing the cysticercus larvae [27]. Besides, as majority of the respondents were farmers before imprisonment, they might had been in contact with contaminated soil and thus acquired hookworm infections.

Bivariate and multivariable analyses were carried out to identify factors associated with IPIs among the participants. As a result, the positivity rate of intestinal parasites was higher among participants who were illiterate (41.7%, 15/36) and who attained primary school (49.5%, 46/93) relative to those who attained secondary school or higher. However, the difference was not statistically significant. Higher prevalence of intestinal parasites among illiterate & primary school participants has been reported elsewhere in Ethiopia [1, 28]. Education has significant impact on the awareness and/or knowledge of the community towards the transmission, prevention and control of infectious diseases. Moreover, the result of the present study revealed that inmates with untrimmed hand fingernails showed significant association with intestinal parasitic infections in bivariate analysis (COR 1.864, 95% CI 1.08–3.20). Untrimmed fingernails are important sources of infections with intestinal parasites as they are difficult to clean. Many of the intestinal protozoa including *E. histolytica/dispar/moshkovskii* & *G. lamblia* and few of the intestinal helminthes (such as *E. vermicularis*) are directly infectious from fresh feces. Hence, infective stages of such parasites can cause external auto-infection to infected inmates when lodged in their fingernails.

Multivariable analysis showed that participants who stayed in the prison for 1 year or less were more likely to acquire intestinal parasitic infections than those who stayed longer (*p* = 0.013). This was in line with a report from Maiduguri prison [24], Nigeria and Bedelle prison [29], Ethiopia. Although this study lacks comparative groups, inmates could have come from areas high risk of intestinal parasitic infections. Moreover, participants who stayed longer in the prison might have been treated against intestinal parasites earlier to the study and not re-infected.

### Limitations

One of the main limitations of the present study is that it was difficult to differentiate the Entamoeba species identified among the participants as to whether they were the non-invasive type *Entamoeba dispar/moshkovskii* or the potentially invasive species, *Entamoeba histolytica*. This was because of unavailability of laboratories that differentiate the Entamoeba species in our set up. Furthermore, food handlers in the prisoners’ cafeteria, which could be potential sources of parasite infections, were not assessed to implicate as a risk factor for the presence of intestinal parasites among the prisoners. It was because of budget constraint to include these food handlers in the study.

## Conclusions

This study has indicated that 42.6% of the study participants were infected with intestinal parasites. *E. histolytica/dispar/moshkovskii* was the predominant parasite identified among the inmates. In addition, intestinal protozoa were more prevalent than intestinal helminthes. This all indicate poor hygienic conditions in the prison services. Therefore, regular laboratory examinations with timely treatment, health education on the transmission and prevention of intestinal parasitic infections, would significantly contribute towards improving the health condition of the inmates. On the other hand, further research is imperative to assess the sanitary conditions of the prison services (water supply, kitchen rooms, the living cells etc) and the status, knowledge, and practice of food handlers in the prison.

## Abbreviations

CI: Confidence Interval
COR: Crude Odds Ratio
HIV: Human Immune-deficiency Virus
IPIs: Intestinal Parasitic Infections
SSA: Sub-Saharan Africa
STHs: Soil-Transmitted Helminthes
WHO: World Health Organization
